# Cardiomyocyte Pdk4 response is associated with metabolic maladaptation in aging

**DOI:** 10.1111/acel.13800

**Published:** 2023-02-16

**Authors:** Mohammad Kasim Fatmi, Di Ren, Julia Fedorova, Linda Ines Zoungrana, Hao Wang, Kayla Davitt, Zehui Li, Migdalia Iglesias, Edward J. Lesnefsky, Meredith Krause‐Hauch, Ji Li

**Affiliations:** ^1^ Department of Surgery Morsani College of Medicine Tampa Florida USA; ^2^ Department of Medical Engineering, College of Engineering and Morsani College of Medicine University of South Florida Tampa Florida USA; ^3^ Pauley Heart Center, Division of Cardiology, Department of Internal Medicine Virginia Commonwealth University Richmond Virginia USA; ^4^ Cardiology Section, Medical Service, Richmond Department of Veterans Affairs Medical Center Richmond Virginia USA; ^5^ James A. Haley Veterans' Hospital Tampa Florida USA

**Keywords:** aging, glycolytic activity, Pdk4, transcriptional analysis

## Abstract

Ischemic heart disease (IHD) is the leading cause of death, with age range being the primary factor for development. The mechanisms by which aging increases vulnerability to ischemic insult are not well understood. We aim to use single‐cell RNA sequencing to discover transcriptional differences in various cell types between aged and young mice, which may contribute to aged‐related vulnerability to ischemic insult. Utilizing 10× Genomics Single‐Cell RNA sequencing, we were able to complete bioinformatic analysis to identity novel differential gene expression. During the analysis of our collected samples, we detected Pyruvate Dehydrogenase Kinase 4 (Pdk4) expression to be remarkably differentially expressed. Particularly in cardiomyocyte cell populations, Pdk4 was found to be significantly upregulated in the young mouse population compared to the aged mice under ischemic/reperfusion conditions. Pdk4 is responsible for inhibiting the enzyme pyruvate dehydrogenase, resulting in the regulation of glucose metabolism. Due to decreased Pdk4 expression in aged cardiomyocytes, there may be an increased reliance on glucose oxidization for energy. Through biochemical metabolomics analysis, it was observed that there is a greater abundance of pyruvate in young hearts in contrast to their aged counterparts, indicating less glycolytic activity. We believe that Pdk4 response provides valuable insight towards mechanisms that allow for the young heart to handle ischemic insult stress more effectively than the aged heart.

## Graphical abstract

Cardiomyocyte Pdk4 modulating pyruvate dehydrogenase is impaired in response to ischemic stress in aging.

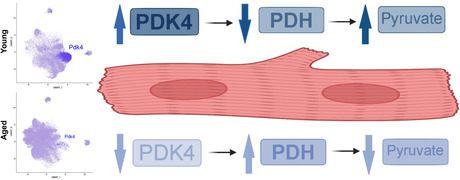

**Abbreviations**
CPT1β: Carnitine palmitoyl transferase 1β
CVD: Cardiovascular disease
I/R: Ischemia and reperfusion
IHD: Ischemic heart disease
LCAD: Long‐chain acyl‐CoA dehydrogenase
MCAD: Medium‐chain acyl‐CoA dehydrogenases
NAD^+^
: Nicotinamide adenine dinucleotide
OXPHOS: Oxidative phosphorylation
PDH E1α: Pyruvate dehydrogenase E1α
PGC‐1α: PPAR gamma coactivator 1‐alpha
PPARα: Peroxisome proliferator activated receptor alpha
TCA: Tricarboxylic acid

## 1 INTRODUCTION

Cardiovascular disease (CVD) remains as the worldwide leading cause of death, particularly, ischemic heart disease (IHD) or coronary artery disease (CAD) is the most prevalent (Tsao et al., 2022). IHD is best described as a chronic plaque buildup in the coronary artery eventually causing ischemic conditions in the heart, leading to tissue death and metabolic alterations (Jiang et al., 2021). This chronic buildup of plaque leads to greater IHD incidence and death increasing with age (Khan et al., 2020). However, the mechanisms that protect the young heart from ischemic stress are not well understood. Various studies have indicated that the aged heart is not only more vulnerable to IHD but also less equipped to handle the stress of ischemic conditions (Ren et al., 2022).

Single‐cell RNA sequencing (scRNA‐seq) has become an increasingly popular method of genetic sequencing since its founding (Hwang et al., 2018). The value of scRNA‐seq comes in the manner of classifying the sequenced data. In contrast to bulk RNA sequencing, scRNA‐seq allows us to observe differences in cell types versus blanket transcriptional differences (Hwang et al., 2018). In our study, we aim to utilize modern sequencing technology to find transcriptional differences between young and aged hearts under healthy physiology and under ischemia/reperfusion (I/R) conditions. These transcriptional differences in the heart may provide insight to differing adaptive responses caused by aging in the form of gene regulation. We will investigate four common cell types found in cardiac tissue: cardiomyocyte, endothelial cells, fibroblast, and macrophage. We aim to discover novel gene regulation under ischemic conditions in the various cell types that may provide clues to understanding the effects of aging.

## 2 RESULTS

### 2.1 Integration and cell types

**FIGURE 1:**
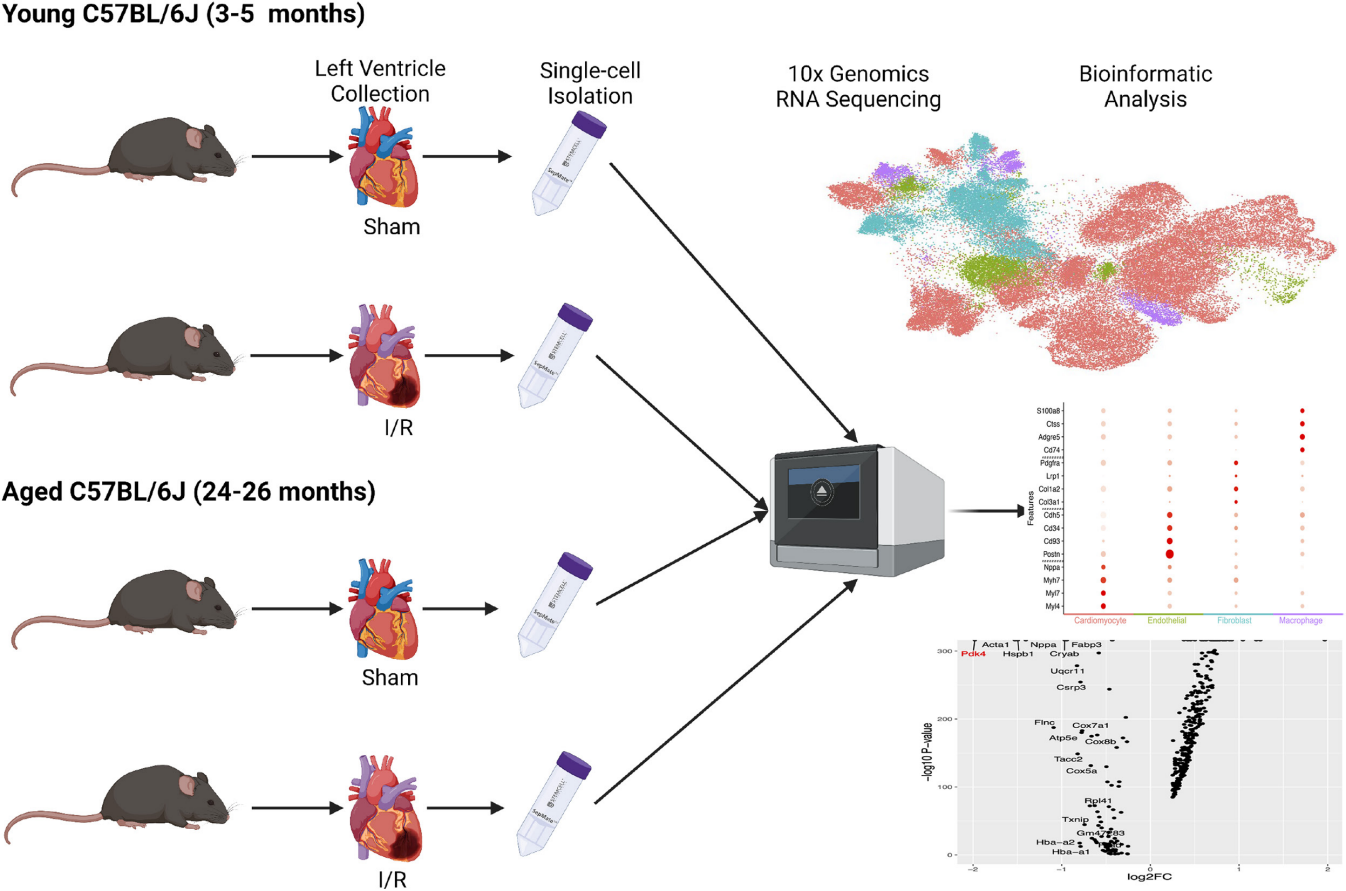
Graphical synopsis of experimental design. We isolated left ventricle tissue from young (3–5 months) and aged (24–26 months) C57BL/6J mice under sham operations or 45 min of ischemia/24‐h of reperfusion (I/R) conditions. Once single‐cell suspensions were achieved, cells were prepared for sequencing using the 10× Chromium system.

**FIGURE 2:**
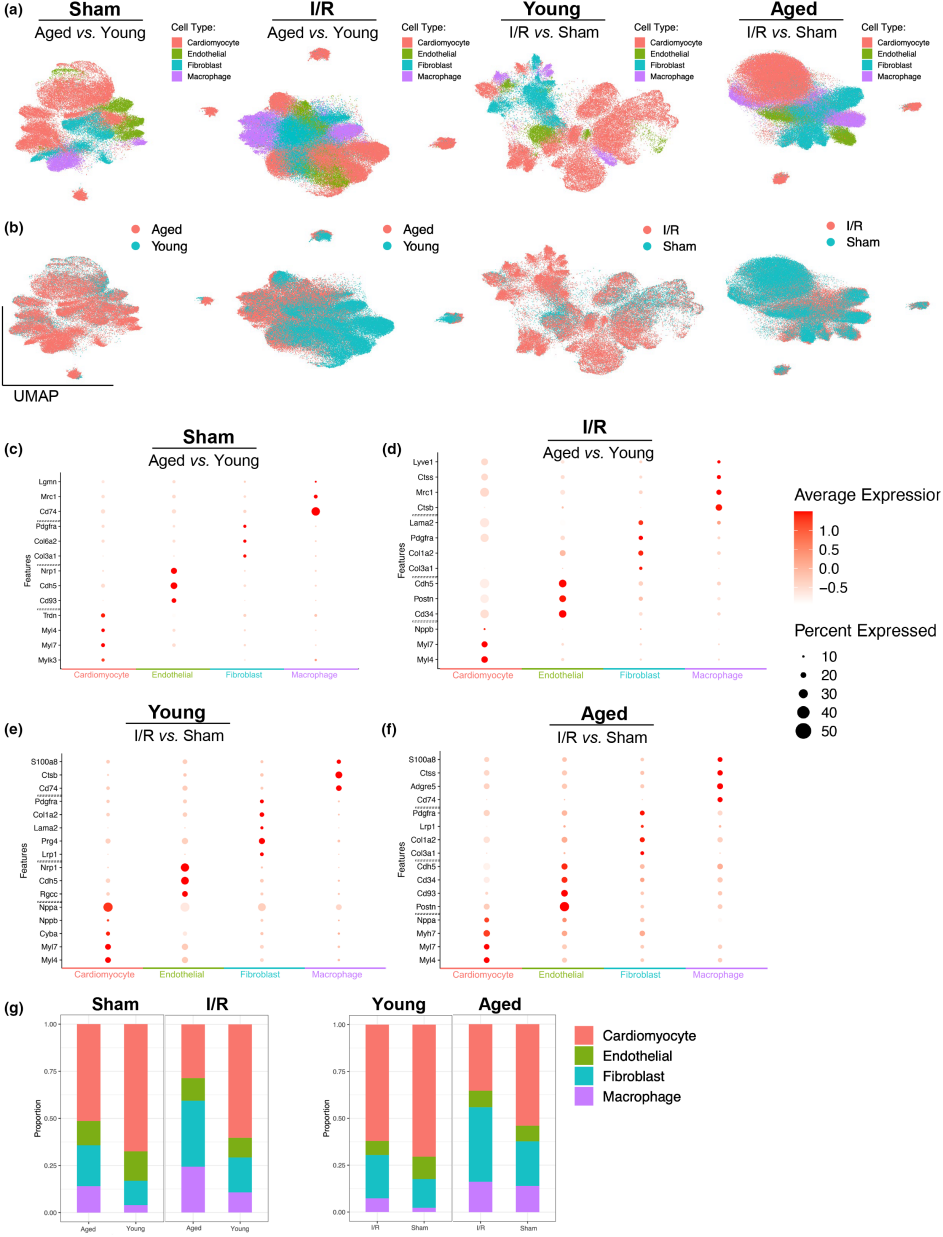
(a) UMAP dimensional projection of each integrated dataset, clusters colored by cell‐type manually identified by marker genes. (b) Matching UMAP dimensional projections, clusters colored by sample age. (c–f) Corresponding dot plots for each integrated sample depicting key marker genes used for manual cell‐type identification. Displays cluster gene expression level compared to global expression in assay (color) and gene expression level within each specific cell‐type cluster (size). (g) Proportion of each cell‐type observed in each integrated dataset.

Our comparative analysis allowed us to observe transcriptional differences in young to aged C57BL/6J wildtype mice. Four RNA samples were received for analysis that allowed for our integrated bioinformatic analysis (Figure 1). Observing the effects of ischemic insult, we compared young I/R to young sham groups and aged I/R to aged sham groups. Additionally, comparison of aged sham to young sham and aged I/R to young I/R were done to investigate the effects of aging (Figure 2a). Following the integration vignette from the Satija Laboratory (Tim Stuart et al., 2019), the maximum overlap of each sample was achieved (Figure 2b). Vital to our study, integration ensured when each cluster was annotated with cell‐type, it contained cells from both samples that form each dataset.

Determining that manually annotating cell types led to the most accurate and consistent data in our datasets, multiple marker genes were found that allowed for annotations (Figures 1f and 2c). Integration established largely consistent marker genes for each cell‐type throughout our datasets. Cardiomyocyte marker genes *Myl4* and *Myl7* were observed in all four datasets, likewise *Nppa*/*Nppb* were profound datasets except for the aged and young sham comparison. The aged and young sham dataset expressed two unique cardiomyocyte marker genes, *Trdn* and *Mylk3*, were strongly expressed and used for annotation for this comparison (Figure 2e).

Across all groups, the most consistent marker gene for endothelial cells was *Cdh5*; notably, each dataset expressed unique endothelial marker genes. Exclusive to the young I/R and sham comparison, Rgcc was significantly expressed and used for endothelial cell annotation (Figure 2c). Furthermore, *Nrp1* was found significantly expressed in the young I/R and sham comparison along with the aged and young sham comparison and used as a marker gene (Figure 2c,e). *Cd94* and *Cd34* were used as endothelial cell markers in the aged I/R and sham, aged and young sham, and aged and young I/R comparisons (Figure 2d,f). Lastly, *Postn* was only remarkably expressed in the aged I/R and sham comparison and in the aged and young I/R comparison (Figure 2d,f).

Annotating fibroblast cells also revealed generally consistent marker genes with some exceptions. *Pdgfra* was remarkably expressed in all datasets and widely regarded as a strong fibroblast marker gene. Collagen‐type genes: *Col1a1*, *Col1a2*, *Col3a1*, and *Col6a2* were also found throughout the datasets and were used to annotate fibroblast cells (Figure 2c–f). In the young I/R and sham comparison, *Prg4* was uniquely identified as significantly expressed as used as a fibroblast marker gene (Figure 2c).

Finally, annotating macrophage cell populations required further analysis. The marker genes identified throughout all datasets were *Cd74* and *Ctss/Ctsb*, while *S100a8* and *Mrc1* were found in most datasets and used to annotate macrophage cells (Figure 2c–f). The aged and young sham comparison independently expressed *Lgmn* (Figure 2e), and the aged and young I/R comparison solely expressed *Lyve1* (Figure 2f). Altogether, this differing expression of marker genes in cell populations indicates shifting cell states and expression profiles and indicates preliminary cell variation in response to their respective conditions.

Moreover, the proportion of each cell‐type is also a major indicator of intriguing changes in cell populations (Figure 2g). We observed greater cardiomyocyte cell populations under sham operations and in young mice. Expectedly, larger cell populations of fibroblast and macrophage cells existed under I/R conditions regardless of age. While cardiomyocyte cells are more abundant in our young sample, independent of condition, the aged sample contains more fibroblast and macrophage cells. However, throughout all our testing datasets, the endothelial cell populations do not drastically shift despite change in age or stress. The fluctuations in cardiomyocyte populations occurring strikingly in response to ischemic stress in aging inspired us to further investigate transcriptional differences in cardiomyocytes.

### 2.2 Differential expression and enrichment

**FIGURE 3:**
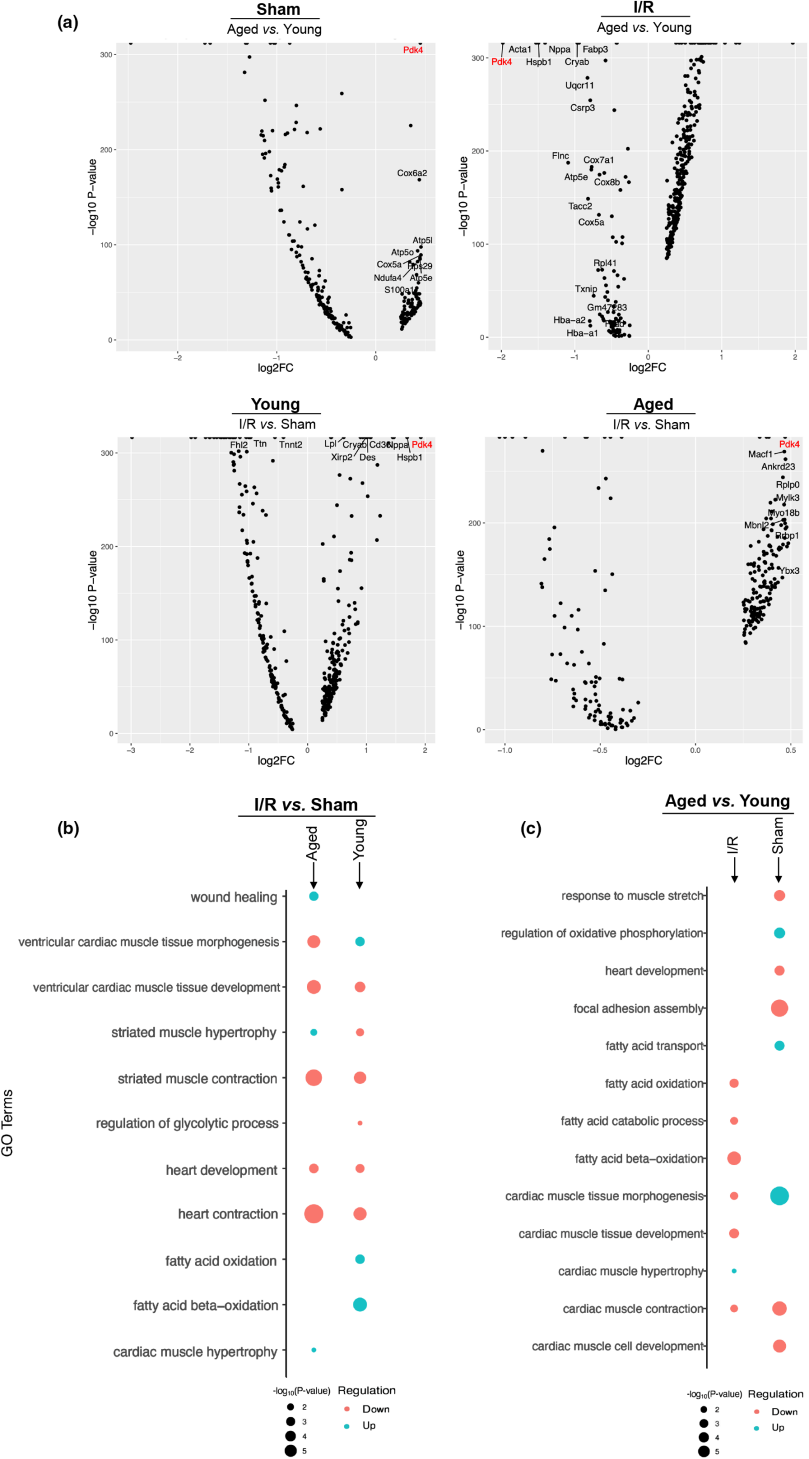
Differential Expression Testing. (a) Differentially expressed features in cardiomyocyte cell populations for all comparative datasets showing gene regulation based on foldchange (Positive is upregulated and negative is downregulated in testing variable). All genes presented received an adjusted *p*‐value of >0.05 from *Seurat* and plotted with −log_10_ for visualization. Gene Ontology (GO) Biological Process Enrichment. (b) Enriched terms from Aged and Young I/R versus Sham cardiomyocyte integrated datasets. Testing variable is I/R condition (i.e., heart contraction under I/R conditions is downregulated in aged and young samples compared to sham operations). (c) Enriched terms from I/R and Sham Aged versus Young cardiomyocyte integrated datasets. Testing variable is Aged condition (i.e., cardiac muscle tissue morphogenesis in the aged sample is downregulated under I/R conditions and upregulated under Sham operations compared to young sample). All enriched terms received an adjusted *p*‐value of >0.05 from EnrichR and plotted with −log_10_ for visualization.

Integration established stable cell types between samples allowing for analyzing differentially expressed genes (DEGs) in specific cell types between two samples—for example, comparing gene regulation in fibroblast cells between I/R and sham conditions. In differential expression testing, cardiomyocyte cell populations were targeted to compare gene (feature) regulation between conditions (Figure 3a). The data from this test was used to generate our Gene Ontology (GO) biological process enrichment data.

Our enrichment data are split into our I/R and sham data (Figure 3b) and our aged and young data (Figure 3c). Under I/R stress, many biological cardiac processes are downregulated regardless of age including heart and muscle contraction and heart development. Notably, upregulation of fatty acid oxidation (FAO) processes and downregulation of glycolytic processes under I/R stress were observed in young mice (Figure 3b). Interestingly, while there is downregulation of ventricular cardiac tissue morphogenesis and upregulation of striated muscle hypertrophy in aged mice, the opposite was observed in the young mice. Aged mice also exhibited biological processes, such as wound healing, that were not seen in the young mice enrichment (Figure 3b).

In our aged and young dataset (Figure 3c), we recognized that many similar processes that were downregulated in the previous dataset (Figure 3b) were also downregulated in this comparison. Under sham conditions, muscle contraction and development, focal adhesion, and responses to muscle stretch are downregulated in aged mice compared to young mice (Figure 3c). Further, under I/R stress, heart and muscle contraction, development, morphogenesis, and FAO related processes are downregulated in aged mice (Figure 3c). The enrichment data retrieved from our differential expression testing revealed that not only were FAO processes downregulated under ischemic stress conditions compared to sham conditions (Figure 3b) but were also downregulated in aged mice compared to young mice under ischemic stress (Figure 3c). This encouraged us to attempt to reveal individual DEGs that may aide in these metabolic changes associated with our differential expression data.

### 2.3 Identification of pyruvate dehydrogenase kinase 4

**FIGURE 4:**
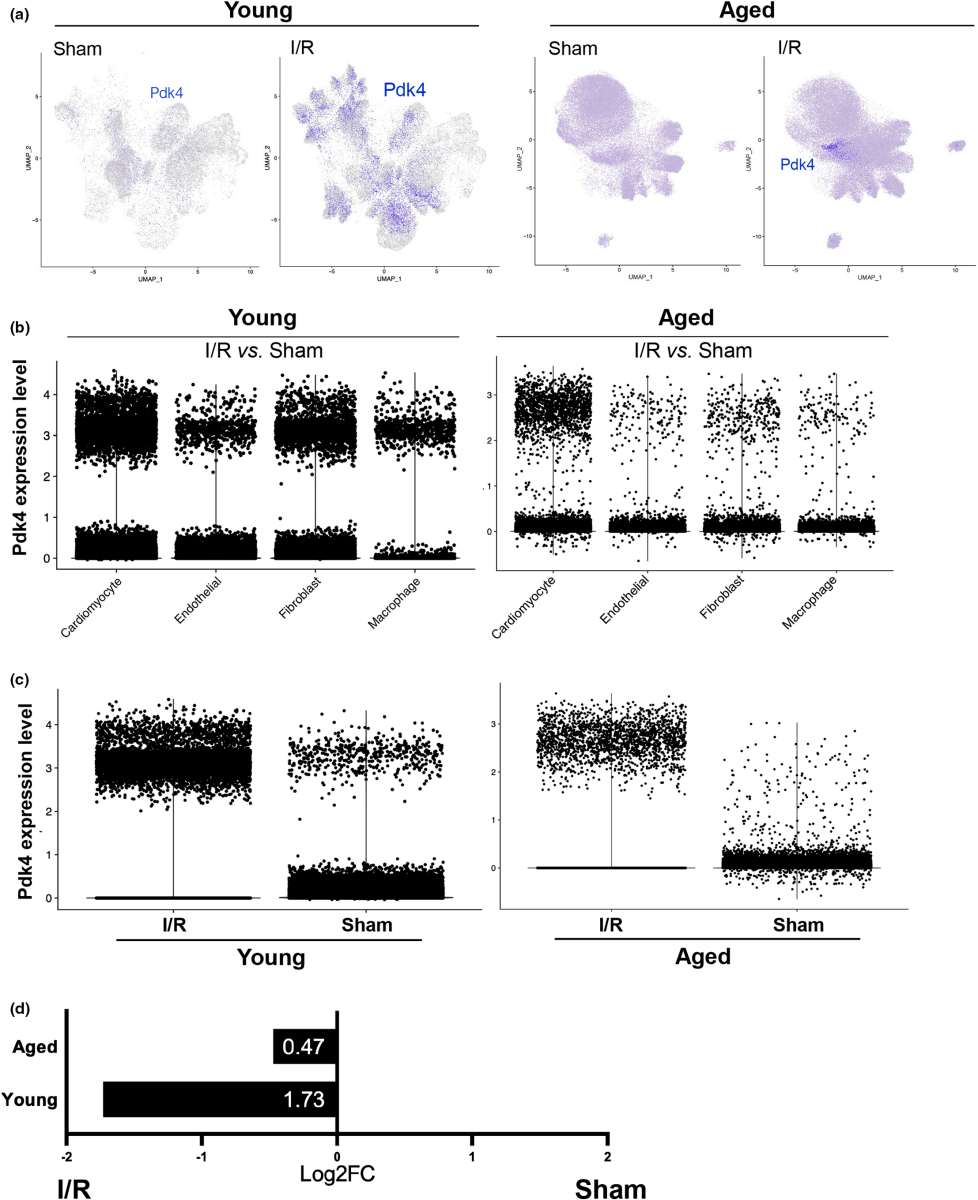
*Pdk4* expression in I/R versus Sham datasets. (a) Split feature plots displaying location and origin of *Pdk4* expression in the UMAP dimensional plot. (b) Expression level plots displaying amount of cells expressing *Pdk4* in each cell‐type. (c) Expression level plots displaying amount of cells expressing *Pdk4* in each sample age and condition. (d) Log2FoldChange statistical expression values of *Pdk4*. *Pdk4* expression values retained a 0 adjusted *p*‐value.

During our study, we identified Pyruvate Dehydrogenase Kinase 4 (*Pdk4*) as a gene that had significant changes in regulation during our differential expression testing (Figure 3a). *Pdk4* is predominantly expressed in cardiomyocytes (Figure 4a,b). While this is expected, as *Pdk4* is well regarded as a strong cardiomyocyte marker gene, the differing expression levels are quite notable. In contrast to sham conditions, *Pdk4* is expressed remarkably after inducing ischemic stress in both aged and young mouse cardiomyocytes (Figure 4c). The relative expression of *Pdk4* in cardiomyocytes is over three times greater in young mice than in aged mice when comparing them to their healthy (sham) counterparts. In the young cardiomyocyte sample, *Pdk4* has a fold‐change value of 1.73 under I/R stress compared to sham and a 0.47‐fold‐change value in the same cardiomyocyte feature expression test (Figure 4d).

**FIGURE 5:**
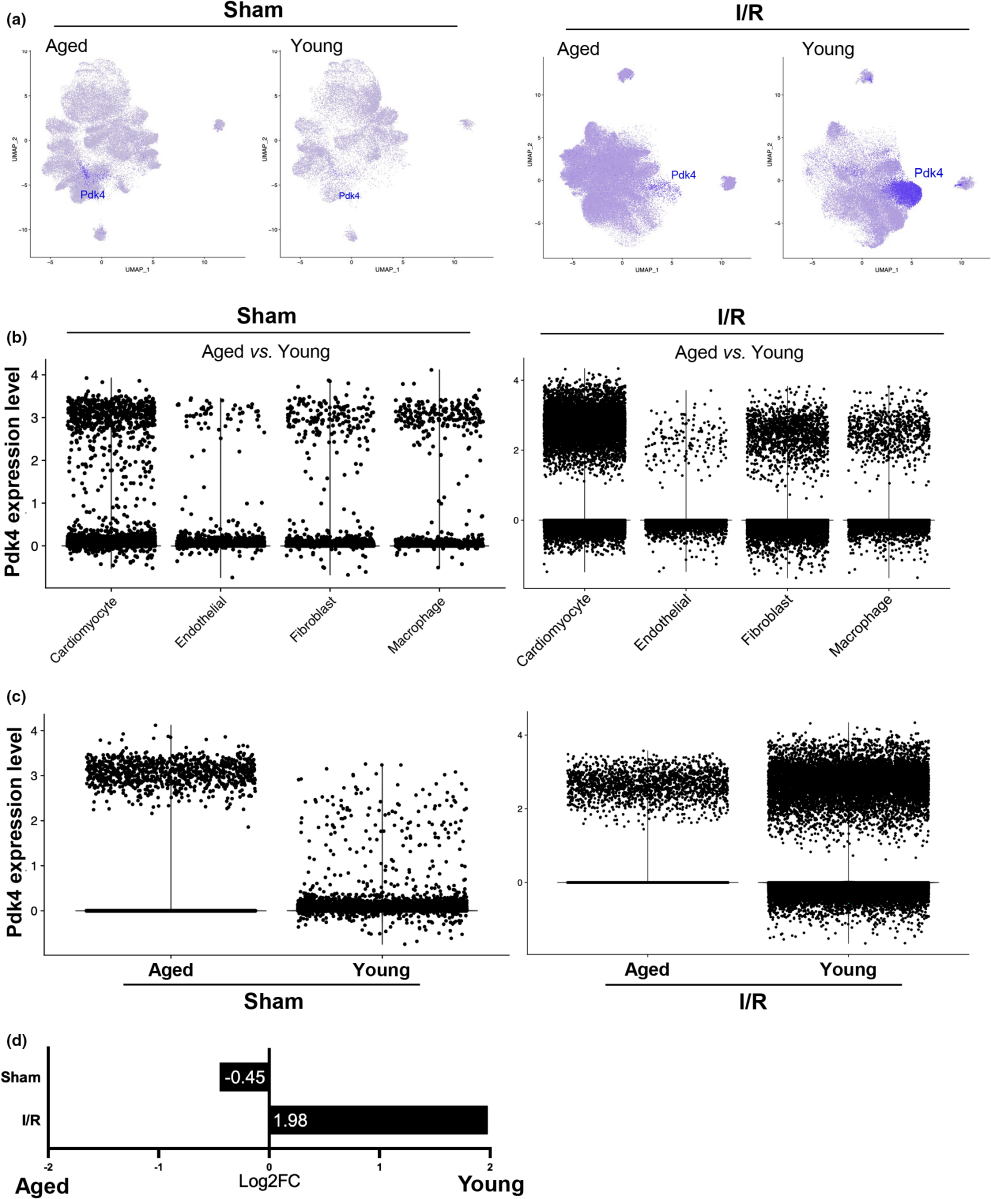
*Pdk4* expression in Aged versus Young datasets. (a) Split feature plots displaying location and origin of *Pdk4* expression in the uniform manifold approximation and projection (UMAP) dimensional plot. (b) Expression level plots displaying of cells expressing *Pdk4* in each cell‐type. (c) Expression level plots displaying amount of cells expressing *Pdk4* in each sample age and condition. (d) Log2FoldChange statistical expression values of *Pdk4*. *Pdk4* expression values retained a 0 adjusted *p*‐value.

While we discovered the expression of *Pdk4* under ischemic stress, the impact of aging on expression required further investigation. Consistent with our previous datasets (Figure 4a,b), Pdk4 was seen predominantly expressed in cardiomyocytes (Figure 5a,b). When comparing aged and young mouse cardiomyocytes, under sham conditions, *Pdk4* is expressed more in aged mice than in young mice. However, under I/R stress, young mice exhibit significantly greater *Pdk4* expression than in aged mouse cardiomyocytes (Figure 5c). This upregulation of *Pdk4* in the aged mice under sham conditions may provide further insight into age‐related gene regulations that occur under physiological conditions. Aged mice under sham conditions express *Pdk4* in cardiomyocytes at a 0.45 fold‐change value when compared to young mouse cardiomyocytes. When under I/R stress, young mouse cardiomyocytes exhibited a fold‐change value of 1.98 when compared to their aged counterparts. This is a preeminent indicator of age‐related expression deficiency that effects the energy source of the heart (Figure 5d).

### 2.4 Alterations in Pdk4 protein levels

**FIGURE 6:**
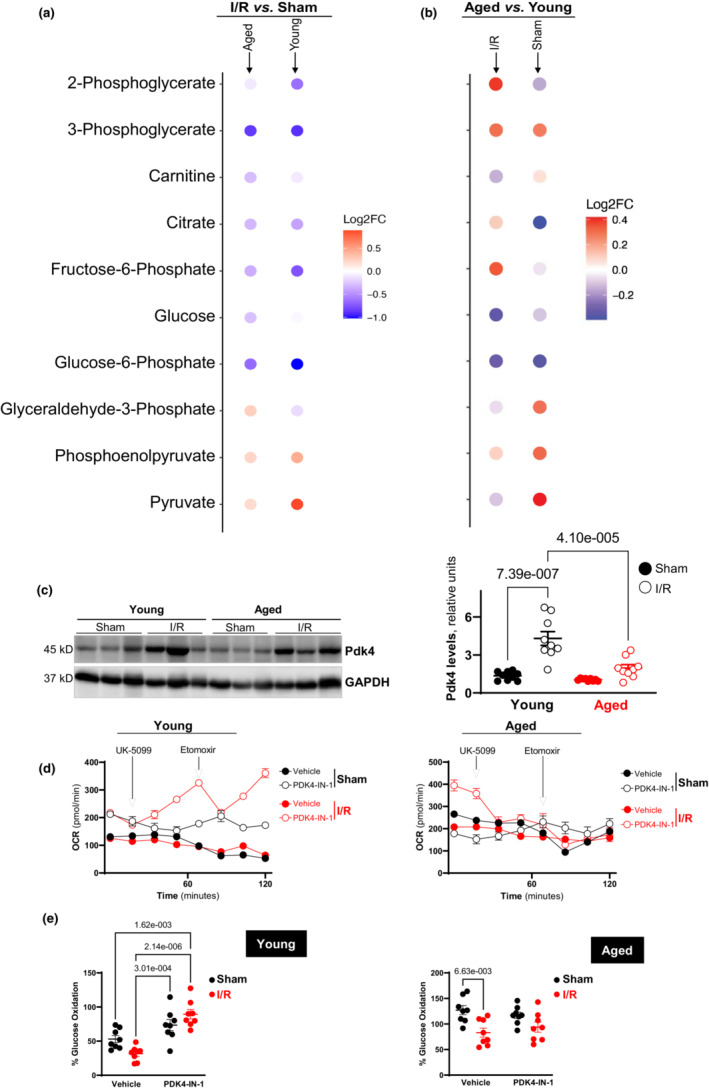
(a) Representative immunoblotting (left) and quantitative analysis (right) of Pdk4 levels in young and aged hearts under sham operations (shaded circles) or I/R conditions (open circles), *n* = 9. (b) Processed LC–MS/MS metabolomic analysis by XCMS and MetSign showing relative abundance of metabolites. All metabolites received a Grubs' two‐tail test *p*‐value of >0.05. Metabolite abundance in response to I/R stress compared to sham conditions in aged and young mice. (c) Metabolite abundance comparing aged to young mice under I/R and sham conditions. (d) The oxygen consumption rate (OCR) and (e) the glucose oxidation dependency measured by Seahorse XF analyzer in young/aged cardiomyocytes under sham operations or I/R conditions with or without Pdk4 inhibitor, PDK4‐IN‐1, *n* = 8.

Taking advantage of immunoblotting, we measured *Pdk4* protein content. We found that protein levels of *Pdk4* matched trends observed in transcription. *Pdk4* protein level was significantly increased in young hearts under I/R conditions versus sham operations (Figure 6a). Though *Pdk4* protein level was upregulated, it did not reach statistical significance in aged heart under I/R versus sham (Figure 6a). Moreover, the higher *Pdk4* levels in young versus aged hearts under I/R stress conditions indicate an impairment in *Pdk4* response in aging (Figure 6a). The *Pdk4* protein response in aging did not show significant difference as compared to young heart as *Pdk4* mRNA response from Single‐Cell Seq analysis under sham operations. This suggests that age‐related alterations in *Pdk4* mRNA expression may not contribute to cardiac metabolic regulation in response to I/R pathological stress.

### 2.5 Metabolomic analysis

The metabolomics data allowed for testing of key glucose metabolites and their comparative abundance. *Pdk4* is known to inhibit pyruvate dehydrogenase (PDH) when expressed. We compared the abundance of pyruvate as well as its downstream and upstream metabolites in I/R versus Sham and Aged versus Young conditions (Figure 6b,c).

Following the results obtained from differential expression testing (Figure 4d), we found a greater presence of pyruvate under I/R stress compared to sham conditions in both aged and young mice (Figure 6b). This indicates that with the increased expression of *Pdk4*, pyruvate accumulates in the cytoplasm as it is no longer being metabolized. We observed that most glycolytic upstream metabolites before phosphoenolpyruvate are more abundant in sham conditions in both aged and young mice except for glyceraldehyde‐3‐phosphate which was present in greater quantities under I/R conditions in aged mice (Figure 6b).

We discovered that aged mice contain more pyruvate in their myocytes than young mice under sham conditions (Figure 6c). We could expect this result as transcriptional *Pdk4* expression is slightly upregulated leading to inhibition of PDH causing accumulation of pyruvate. It is improbable that the marginal upregulation of *Pdk4* is the primary cause for the results observed here and further research is needed. We detected that pyruvate is greater in young mice than aged mice under I/R conditions (Figure 6c). Based on our transcriptional data indicating major *Pdk4* expression in young mice (Figure 5d), we expected greater inhibition of PDH in young mice than in aged mice under ischemic conditions. These results strongly propose that *Pdk4* is regulating glucose metabolism in response to stress such as ischemia conditions but is also impacted during the aging process, advancing research in metabolic alterations due to aging.

### 2.6 Glucose oxidation dependency

Finally, a mitochondrial fuel dependency test was performed to characterize the role of *Pdk4* in glucose oxidation of cardiomyocytes under physiological and pathological conditions. The Seahorse XF analyzer paired with a glucose oxidation dependency test was used on isolated cardiomyocytes from both aged and young hearts under sham and I/R conditions. Furthermore, we tested all groups with and without *Pdk4* inhibition. The results show a decrease in glucose oxidation dependency after ischemic insult in both aged and young cardiomyocytes (Figure 6d,e). There were not significant alterations in young cardiomyocytes under sham or I/R conditions (Figure 6e), while a significant difference occurred in aged cardiomyocytes under I/R versus sham (Figure 6e). In addition, we found a significant increase of glucose oxidation dependency in young cardiomyocyte from I/R versus sham with *Pdk4* inhibitor (Figure 6e), indicating a critical role of *Pdk4* in cardiomyocyte glucose metabolism. We did not observe significant difference in glucose oxidation dependency with *Pdk4* inhibitor between young and aged cardiomyocytes from sham operations groups (Figure 6d,e). When comparing glucose oxidation dependency between young and aged cardiomyocytes, the aged versus young cardiomyocytes significantly depend more on glucose oxidation under both sham and I/R conditions (Figure 6e). *Pdk4* inhibitor treatment increases glucose oxidation dependency in young cardiomyocytes but not in aged cardiomyocytes from I/R groups (Figure 6e). Correspondingly, this implies that Pdk4 activation in response to I/R stress as a metabolic adaptive response is impaired in aged hearts as compared to young hearts that could lead to cardiac vulnerability in aging to ischemic insults caused by ischemia and reperfusion.

## 3 DISCUSSION

Understanding the effects of aging remains to be an important objective in clinical medicine, especially pertaining to IHD. The mechanisms by which cardiac cells adjust and communicate during injury are still unknown. To uncover underlying transcriptional differences in aged and young mouse cells, we employed scRNA‐seq. Identification of novel DEGs provide insight towards inadequately understood shifts in adaptive responses to ischemic stress between the aged and young heart. Targeting the genes responsible for metabolic deficiency in the aged heart may facilitate therapies and potential treatments for the injured heart.

To best investigate the pathophysiology of IHD, a murine model for myocardial infarction was utilized. The 45‐min ischemia, 24‐h reperfusion period prior to sequencing causes physiological symptoms analogous to myocardial infarction and is an accepted method for simulating short‐term I/R injury (Xu et al., 2014). Our previous research using this experimental method has shown that infarct area significantly increases in aged I/R mice compared to young I/R mice (Wang et al., 2018). Aged murine hearts after I/R conditions exhibit critically lower ejection fraction and fractional shortening compared to their young counterparts (Wang et al., 2018). The investigation of resulting adverse downstream effects was motive for this study.

Single‐cell RNA sequencing was employed to accomplish the study of transcriptional differences in specific cell types. Utilizing the integration vignette in *Seurat*, we completed a comparative analysis between I/R and sham conditions as well as between aged and young mice. After identifying cell types, we enriched our differential expression data to provide insight into adverse biological processes. We found FAO‐related processes to be upregulated in young mice and downregulated in aged mice under ischemic stress.

Following the enrichment data, we sought to discover individual significant DEGs. This process uncovered notable changes in the expression of *Pdk4* in cardiomyocytes which critically regulates pyruvate dehydrogenase (PDH) (Chambers et al., 2011; Shuai Zhang et al., 2014). Metabolic remodeling is a well‐known consequence increased stress such as ischemia in the heart (Khan et al., 2020; Wu et al., 2017). In contrast, the young heart retains a stronger ability to recover from ischemic insult (Zhang et al., 2021). Based on our data, we propose that the aged heart under ischemic stress relies on inferior glucose oxidation as the heart's main energy source versus other energy‐rich metabolic pathways.

Immunoblotting studies to observe the protein content of *Pdk4* were performed to corroborate the RNA‐seq findings. The results from immunoblotting support the increased expression of *Pdk4* after ischemic insult in young mice. While we observed an upregulation of *Pdk4* expression in aged mice, that result was not found to be significant. Notably, there was a significant increase of *Pdk4* expression between young and aged mice following an ischemic insult. There was no significant difference in protein level between young and aged sham mice, the transcriptional *Pdk4* upregulation in aged sham mice was modest, and thus, a significant change in the protein content was not expected. Though there was an average increased protein level in aged I/R compared to aged sham mice, this finding was also not considered significant. We consider due to the diminished transcriptional expression of *Pdk4* in aged I/R mice, the resulting protein level expression would not be great enough to reach significance when compared to sham conditions.

Encountering this change in an essential metabolite regulator on both transcriptional and protein level, we decided to use biochemical metabolomic data to test the abundance of key glycolytic metabolites. The metabolomic data were consistent with the previous expression analyses. The expression of *Pdk4* in our single‐cell comparative groups match the abundance of metabolites in the metabolomic comparative groups. Principally, the abundance of pyruvate is greater under ischemic stress regardless of age, this finding correlates with the *Pdk4* expression findings, the higher transcriptional expression of *Pdk4* under ischemic stress indicates inhibition of PDH leading to accumulation of pyruvate in the cell. Although other isoforms of pyruvate dehydrogenase kinase exist and regulate PDH, *Pdk4* has been found to be the more compelling regulator of glucose oxidization and resulting PDH (Tao et al., 2013).

The expression pattern in our single‐cell data indicates that *Pdk4* has greater expression in aged mice under sham conditions. Furthermore, we found that pyruvate is more abundance in aged mice than young mice under sham conditions. While this supports the scRNA‐seq data and that *Pdk4* is a major glycolytic regulator in the heart (Liu et al., 2017), it is unlikely that the slight transcriptional upregulation of *Pdk4* under sham conditions lead to the results observed by metabolomics. We believe other signaling pathways may be involved as aging leads to impaired mitochondrial function that decreases oxidative phosphorylation and increases the production of reactive oxygen species (Chen et al., 2020; Lesnefsky et al., 2016). It is intriguing to speculate that the increased transcriptional expression of *Pdk4* in the aged heart in the baseline state may represent an adaptive response to the age‐induced mitochondrial dysfunction. Notably, recent attempts to downregulate *Pdk4* to mitigate the effects of cardiac aging have been successful in baseline conditions signifying the link between *Pdk4* and aging in normal physiology (Zhang et al., 2022). Under I/R stress, there is a larger abundance of pyruvate in the young mice than in the aged mice, consistent with transcriptional expression of *Pdk4* being higher in young mice.

It is understood that increased glucose oxidation can be beneficial during ischemic conditions (Lopaschuk & Stanley, 1997; Tran & Wang, 2019); a chronic deficiency of other metabolic pathways is generally detrimental (Sithara & Drosatos, 2021). The expression pattern of *Pdk4* observed suggests that the aged heart no longer strongly expresses the gene after ischemic insult. Seemingly, the aged heart suffers from the loss of flexibility to utilize other sources of energy compared to the young heart. Our enrichment data indicate that the aged heart has a decreased capacity for FAO under stress while the young heart upregulates FAO, consistent with findings of Pdk4 expression recognized as marker for increased FAO (Pettersen et al., 2019). We propose that *Pdk4* is linked to this deficiency in metabolic programming that occurs with aging.

Accordingly, employing the Seahorse XF analyzer we were able to test the glucose oxidation dependency of cardiomyocytes under sham and I/R conditions. The observed results were congruent with the previous experimentation. Particularly, the decrease in glucose oxidation dependency following the ischemic insult in aged mice adhere to the increase of *Pdk4* observed transcriptionally and on the protein level. When statistically comparing aged to young glucose dependency, significant findings of increased glucose dependency in aged cardiomyocytes under both sham and I/R conditions were observed which is consistent with past research investigating metabolism in the aged heart (Sithara & Drosatos, 2021). Inhibiting *Pdk4* in vitro resulted in significant increases in glucose oxidation after ischemic insult in young cardiomyocytes compared to baseline as well as between aged and young cardiomyocytes under sham conditions. Remarkably, with *Pdk4* inhibited, young cardiomyocyte glucose oxidation was increased to the point of not being significantly different than aged cardiomyocytes under I/R conditions; therefore, indicating the role of *Pdk4* on cardiomyocyte metabolism during ischemic insult.

Our results reveal that *Pdk4* expression is linked to energy sources that alter with age in the heart after ischemic insult. Not only does vulnerability to IHD increase with age but the inability to adapt to the stress of ischemia is an additional consequence of aging (Zhang et al., 2021). Deficiency of *Pdk4* in the aged heart has been observed in the past (Hyyti et al., 2010); its role following I/R indicates metabolic alterations that may contribute to increased injury compared to the young heart.

In the aging heart, mitochondria suffer additional damage with ischemia that is superimposed upon age‐induced defects, enhancing myocardial injury during the early reperfusion period (Lesnefsky et al., 2016, 2017; Mohsin et al., 2019). It is possible that the baseline increase in *Pdk4* expression in the adult heart represents a compensatory attempt to regulate the metabolism of these damaged mitochondria that are injurious to the heart. In response to I/R stress, *Pdk4* is less robustly expressed in the aged heart. Suggesting that during reperfusion, metabolism in mitochondria that contain both aging and ischemic defects is restrained, potentially representing a mechanism that is a result of greater injury that occurs in the aged heart following I/R. Increased FAO results in higher stress on mitochondria (Menendez‐Montes et al., 2021), these damaged cardiomyocytes are likely unable to handle the increased stress like their young counterparts. Further investigation regarding the young heart inhibiting PDH after ischemic conditions is required to better understand our results. We plan to further investigate *Pdk4* and the relationship it has with age‐related stress and possible therapeutic strategies impacting the metabolic remodeling in the aged heart.

Although we identified *Pdk4* as a significantly differentially expressed gene between aged and young mice, an individual gene cannot convey the entire adaptive response of the young heart to ischemic stress. While left ventricular samples were collected from four populations of both aged and young mice, we only collected I/R samples after 45 min of ischemia and 24‐h reperfusion. Collecting sequential samples throughout and following the reperfusion period would allow for more accurate expression testing of differentially expressed genes. Other studies have found that high *Pdk4* expression as a physiological response in skeletal muscle following prolonged exercise typically diminish during rest (Pilegaard & Neufer, 2004). Our findings show that *Pdk4* in the left ventricle remains robustly expressed following 24 h of reperfusion. The long‐term pathological expression of *Pdk4* will provide considerable insight towards the heart's recovery from ischemic insult. Additionally, observing expression over time would permit us to discover other genes that follow the *Pdk4* expression pattern revealed in our study.

In our study, we performed metabolomic analysis, immunoblotting, and in vitro metabolism testing. In future studies, it would be beneficial to include transgenic mice that have *Pdk4* knocked out in cardiomyocytes and mice with *Pdk4* overexpressed. While global knockout *Pdk4* transgenic mice have been observed to increase glucose oxidation (Jeoung & Harris, 2008), we believe investigating cardiomyocyte‐specific knockout in vivo would be the most beneficial. Cardiomyocyte‐specific *Pdk4* knockout mice have been generally found to have favorable outcomes under normal physiology (Cardoso et al., 2020), additional investigation of these mice under I/R conditions is essential to future studies. Furthermore, we plan to obtain *Pdk4* overexpressed transgenic mice to observe effects on metabolism and heart function after ischemic insult as previous research indicates overexpression leads to increased fatty acid catabolism and without detriment (Chambers et al., 2011; Zhao et al., 2008).

While this study used a mouse model, it would be beneficial to test the expression of *Pdk4* in the human heart to provide important insight into metabolic alterations present in humans with aging. Additionally, though cardiomyocytes are vital cells that are impacted by ischemic stress, we aim to target more differentially expressed genes in other cell types in the future. In our scRNA‐seq data, we identified cardiomyocytes, endothelial cells, fibroblasts, and macrophages. Observing transcriptional differences in these other cell types has the potential to further uncover increased adaptive responses in the young heart to ischemic stress.

## 4 EXPERIMENTAL PROCEDURES

### 4.1 Single‐cell RNA sequencing

Young (3–5 months old) and aged (24–26 months old) wildtype (WT) C57BL/6J mice were supplied from NIA contracted Charles River Laboratories (Wilmington, MA). Mice were subjected to 45 minutes of ischemia in LAD surgery and allowed 24 h for reperfusion in both young and aged groups. The heart was collected, and left ventricle separated, the entire left ventricle tissue from both sham and I/R mice were dissociated by the 10× Genomics Chromium Next GEM Single Cell Library and Gel Bead Kit prior to sequencing. Single‐cell samples were sequenced by Genewiz and were received as raw FASTQ files encoded with Sanger/Illumina 1.9 with quality scores above 28 using FastQC 0.11.5 (Babraham Bioinformatics). The raw FASTQ files were subject to 10× Genomics Cellranger application to sort the sequencing data using the mm10 reference genome from 10× Genomics to generate matrix files for bioinformatic analysis. The Raw and Processed RNA‐seq data available from Gene Expression Omnibus (GEO) under accession number GSE213080.

### 4.2 Bioinformatic analysis: Integration

Data analysis was completed by using *Seurat* 4.0 (Yuhan Hao et al., 2021) in *Rstudio* 2.3 IDE (RStudio Team, 2020). Following published vignettes and parameters for sample integration and processing, comparative datasets were generated for analysis (Tim Stuart et al., 2019). The following comparative datasets were made for analysis: Young I/R versus Sham, Aged I/R versus Sham, Aged versus Young Sham, and Aged versus Young I/R were. All comparison datasets were processed using the *Seurat* 4.0 R‐package.

### 4.3 Bioinformatic analysis: Clustering and cell‐type identification

In all our integrated datasets, we scaled the data and performed a PCA reduction with 10 principal components prior to UMAP dimensional reduction using all 10 principal components from PCA reduction. The *FindClusters()* function was used to identify clusters and the resolution was adjusted to generate ~50 clusters. Utilizing the *FindAllMarkers()* function in *Seurat*, we identified marker genes in each cluster to annotate cell types. We subset all clusters with remarkable cell‐type specific marker genes (Log2FC‐value < 2, *p*‐value > 0.05) to allow for cell‐type annotation into four essential cell types: cardiomyocyte, endothelial, fibroblast, and macrophage. To verify the accuracy of our marker genes, we employed the *Human Protein Atlas* (Uhlén et al., 2015) as a genome directory to cross‐analyze our markers to associated cell types in the atlas.

### 4.4 Bioinformatic analysis: Differential expression and enrichment

Once all four comparative datasets had identified cell types, we followed the *Seurat* vignette for differential expression testing. In our testing parameters, the two samples that form each dataset were compared with each cell‐type isolated. Particularly, the RNA data assay is used to generate the data in comparison with using the integrated assay. The RNA assay in contrast to the integrated assay retains the original expression profiles of cells prior to “correction” that occurs during the integration protocol. The original expression profiles of the cells were set as the identities in the *FindMarkers()* function with cell‐type set as the subset identity. We omitted all genes that produced an adjusted *p*‐value greater than 0.05 from all further downstream analysis. These data were used to generate pathway enrichment using the *EnrichR* R‐package (Chen et al., 2013; Kuleshov et al., 2016) and Gene Ontology (GO) term biological processes (Ashburner et al., 2000; Gene Ontology, 2021). The statistical expression values (Log2FC) of *Pdk4* were taken from the differential expression testing, and related plots were created using the ggplot2 R‐package to visualize the expression data (Wickham, 2016).

### 4.5 Immunoblotting

Protein samples were prepared by homogenization of left ventricular tissue mixed with lysis buffer (20 mM Tris–HCl, 137 mM NaCl, 0.5% NP‐40, 0.5 mM 1,4‐Dithiothretiol, Complete protease inhibitor cocktail [Roche], and phosphatase inhibitor cocktail [Sigma]). Protein concentrations were measured by utilizing Bradford assay kit (Bio‐Rad), and proteins were resolved in 10% acrylamide SDS‐PAGE gels then transferred onto polyvinylidene difluoride membranes (Millipore). The primary antibody used for immunoblotting was rabbit polyclonal antibody against Pdk4 (Thermo‐Fischer, Cat #PA5‐102685). All antibodies were used in accordance with manufacture protocol. Relative quantity was calculated by dividing the relative amount of Pdk4 by the relative amount of GAPDH using Bio‐Rad Image Lab 6.0.1 (Bio‐Rad).

### 4.6 Metabolomic biochemical experimentation

2DLC‐MS/MS analysis was completed by first collecting left ventricular tissue samples at 4°C to retain metabolites. Polar metabolites were mixed with methanol and centrifuged to acquire a metabolite supernatant. The supernatant was analyzed by the Thermo Q Exactive HF Hybrid Quadrupole‐Orbitrap Mass Spectrometer coupled to the Thermo DIONEX UltiMate 3000 HPLC system (Thermo‐Fisher Scientific). Utilizing reverse‐phase chromatography (RPC) and hydrophilic interaction chromatography (HILIC) in the HPLC, each sample was analyzed by 2DLC‐MS. Each sample group were split into six pooled samples and analyzed in both positive and negative modes to obtain the full MS/MS spectra at 20, 40, and 60 eV collision energies.

### 4.7 Metabolomic data analysis

Data analysis was completed using XCMS software for spectrum deconvolution and MetSign software to identify metabolites, peak alignment, normalization, and statistical analysis (Wei et al., 2011, 2012, 2014). We matched our data to our in‐house generated database of ion m/z, MS/MS spectra, and metabolite retention time to identify most metabolites. Unmatched data were analyzed in Themo‐Fisher Compound Discoverer Software 2.0 where metabolites with at least 40% similarity scores were selected. We targeted metabolites associated with glycolysis and plotted the abundance change (fold‐change) data using the ggplot2 R‐package (Wickham, 2016). The metabolomics datasets used in this study are available at the National Metabolomics Data Repository under accession number: 2842.

### 4.8 Cardiomyocyte isolation

We tested isolated cardiomyocytes to best understand metabolism modulation in our testing groups. Heparin IV (Fresenius Kabi AG) was administered by intraperitoneal injection to prevent coagulation during perfusion. Once anesthetized, the mice heart was excised and cannulated by the aorta to be connected to the perfusion apparatus (Radnoti LCC). The heart was perfused at 37°C with perfusion buffer followed by digestion buffer. To isolate cardiomyocytes, the heart was removed from the apparatus and gently minced, the remaining solution was suspended and filtered through 100 μm filter to obtain isolated cardiomyocytes.

### 4.9 Seahorse mitochondria testing

The Seahorse Bioscience XF24 Extracellular Flux Analyzer Kit (Seahorse Bioscience) was used to measure glucose dependency in vitro. The Mito Fuel Flex test was completed after subjecting cardiomyocytes to glucose oxidation pathway inhibition by UK5099 (4 μM) followed by long‐chain fatty acid oxidation inhibition by Etomoxir (2 μM). This order of inhibitors allows for calculation of glucose oxidation dependency. The protocol was modified to test dependency in *Pdk4* inhibited cardiomyocytes. Prior to loading isolated cardiomyocytes to the Seahorse cell‐plate, cardiomyocytes were incubated with 10 μM *Pdk4* inhibitor (MedChem Express, Cat# HY‐135954A) in DMEM medium (Seahorse Bioscience) for 5 min at 37°C. The cell‐plate was also loaded with cardiomyocytes suspended in 10 μM *Pdk4* inhibitor in DMEM medium.

### 4.10 Statistical analysis

Data in *Seurat* were collected by the *FindMarkers()* function. This outputs a dataset containing all differentially expressed genes and their corresponding Log2FC and adjusted *p*‐values. Adjusted *p*‐values are generated in *Seurat* by statistically comparing expression with other genes in the assay and performing Bonferroni correction (Satija et al., 2015). We filtered out all genes containing an adjusted *p*‐value greater than 0.05. Likewise, after using *EnrichR*, it produces a dataset containing all associated GO biological processes and corresponding adjusted *p*‐values. *EnrichR* produces Benjamini–Hochberg adjusted *p*‐values (Chen et al., 2013; Kuleshov et al., 2016). We filtered out all GO terms with an adjusted *p*‐value of 0.05 and selected terms representing metabolic and development alterations in the heart.

In our metabolomics data analysis, the Grubbs' test was used to identify outlier data proceeded with a pairwise two‐tail *t*‐test. To determine significance, metabolites that were present in at least 75% of the pooled samples in each group as well as acquired a *p*‐value of <0.05 from the two‐tailed *t*‐test were selected.

Immunoblotting data were validated by performing a Tukey's one‐way ANOVA statistical test in Prism 9.4.1 (GraphPad Software). The Seahorse dependency values were obtained by averaging normalized oxygen consumption rate (OCR) values from each well of the cell‐plate together and utilizing the following equation:

Dependency % = ([Baseline OCR − Target inhibitor OCR]/[Baseline OCR − All inhibitor OCR]) × 100.

The dependency data were validated by performing a two‐way ANOVA statistical test with Šídák correction in Prism 9.4.1 (GraphPad Software). Statistical significance was determined by *p*‐value < 0.05. All figures are shown with means ± standard error (SEM) if possible.

## AUTHOR CONTRIBUTIONS

M.K. Fatmi, D. Ren, and J. Li designed research. M.K. Fatmi, D. Ren, J. Fedorova, L. Zoungrana, H. Wang, K. Davitt, Z. Li, M. Iglesias, and M. Krause‐Hauch performed research. M.K. Fatmi, J. Fedorova, M. Krause‐Hauch, and J. Li analyzed data; M.K. Fatmi, E.J. Lesnefsky, M. Krause‐Hauch, and J. Li interpreted data. M.K. Fatmi, M. Krause‐Hauch. and J. Li wrote the paper.

## CONFLICT OF INTEREST STATEMENT

The authors declare that they have no conflict of interest.

## Data Availability

The data that support the findings of this study are available on request from the corresponding author.

## References

[acel13800-bib-0001] Ashburner, M. , Ball, C. A. , Blake, J. A. , Botstein, D. , Butler, H. , Cherry, J. M. , Davis, A. P. , Dolinski, K. , Dwight, S. S. , Eppig, J. T. , Harris, M. A. , Hill, D. P. , Issel‐Tarver, L. , Kasarskis, A. , Lewis, S. , Matese, J. C. , Richardson, J. E. , Ringwald, M. , Rubin, G. M. , & Sherlock, G. (2000). Gene ontology: Tool for the unification of biology. The Gene Ontology Consortium. Nature Genetics, 25(1), 25–29. 10.1038/75556 10802651 PMC3037419

[acel13800-bib-0002] Cardoso, A. C. , Lam, N. T. , Savla, J. J. , Nakada, Y. , Pereira, A. H. M. , Elnwasany, A. , Menendez‐Montes, I. , Ensley, E. L. , Petric, U. B. , Sharma, G. , Sherry, A. D. , Malloy, C. R. , Khemtong, C. , Kinter, M. T. , Tan, W. L. W. , Anene‐Nzelu, C. G. , Foo, R. S. , Nguyen, N. U. N. , Li, S. , … Sadek, H. A. (2020). Mitochondrial substrate utilization regulates cardiomyocyte cell cycle progression. Nature Metabolism, 2(2), 167–178. PMC7331943 32617517

[acel13800-bib-0003] Chambers, K. T. , Leone, T. C. , Sambandam, N. , Kovacs, A. , Wagg, C. S. , Lopaschuk, G. D. , Finck, B. N. , & Kelly, D. P. (2011). Chronic inhibition of pyruvate dehydrogenase in heart triggers an adaptive metabolic response. The Journal of Biological Chemistry, 286(13), 11155–11162. 10.1074/jbc.M110.217349 21321124 PMC3064169

[acel13800-bib-0004] Chen, E. Y. , Tan, C. M. , Kou, Y. , Duan, Q. , Wang, Z. , Meirelles, G. V. , Clark, N. R. , & Ma'ayan, A. (2013). Enrichr: Interactive and collaborative HTML5 gene list enrichment analysis tool. BMC Bioinformatics, 14, 128. 10.1186/1471-2105-14-128 23586463 PMC3637064

[acel13800-bib-0005] Chen, Q. , Samidurai, A. , Thompson, J. , Hu, Y. , Das, A. , Willard, B. , & Lesnefsky, E. J. (2020). Endoplasmic reticulum stress‐mediated mitochondrial dysfunction in aged hearts. Biochimica et Biophysica Acta ‐ Molecular Basis of Disease, 1866(11), 165899. 10.1016/j.bbadis.2020.165899 32698045

[acel13800-bib-0006] Gene Ontology Consortium . (2021). The Gene Ontology resource: Enriching a GOld mine. Nucleic Acids Research, 49(D1), D325–D334. 10.1093/nar/gkaa1113 33290552 PMC7779012

[acel13800-bib-0007] Hwang, B. , Lee, J. H. , & Bang, D. (2018). Single‐cell RNA sequencing technologies and bioinformatics pipelines. Experimental & Molecular Medicine, 50(8), 1–14. 10.1038/s12276-018-0071-8 PMC6082860 30089861

[acel13800-bib-0008] Hyyti, O. M. , Ledee, D. , Ning, X. H. , Ge, M. , & Portman, M. A. (2010). Aging impairs myocardial fatty acid and ketone oxidation and modifies cardiac functional and metabolic responses to insulin in mice. American Journal of Physiology. Heart and Circulatory Physiology, 299(3), H868–H875. 10.1152/ajpheart.00931.2009 20601465 PMC2944494

[acel13800-bib-0009] Jeoung, N. H. , & Harris, R. A. (2008). Pyruvate dehydrogenase kinase‐4 deficiency lowers blood glucose and improves glucose tolerance in diet‐induced obese mice. American Journal of Physiology. Endocrinology and Metabolism, 295(1), E46–E54. 10.1152/ajpendo.00536.2007 18430968 PMC2493588

[acel13800-bib-0010] Jiang, M. , Xie, X. , Cao, F. , & Wang, Y. (2021). Mitochondrial metabolism in myocardial remodeling and mechanical unloading: Implications for ischemic heart disease. Frontiers in Cardiovascular Medicine, 8, 789267. 10.3389/fcvm.2021.789267 34957264 PMC8695728

[acel13800-bib-0011] Khan, M. A. , Hashim, M. J. , Mustafa, H. , Baniyas, M. Y. , al Suwaidi, S. K. B. M. , AlKatheeri, R. , Alblooshi, F. M. K. , Almatrooshi, M. E. A. H. , Alzaabi, M. E. H. , al Darmaki, R. S. , & Lootah, S. N. A. H. (2020). Global epidemiology of ischemic heart disease: Results from the global burden of disease study. Cureus, 12(7), e9349. 10.7759/cureus.9349 32742886 PMC7384703

[acel13800-bib-0012] Kuleshov, M. V. , Jones, M. R. , Rouillard, A. D. , Fernandez, N. F. , Duan, Q. , Wang, Z. , Koplev, S. , Jenkins, S. L. , Jagodnik, K. M. , Lachmann, A. , McDermott, M. , Monteiro, C. D. , Gundersen, G. W. , & Ma'ayan, A. (2016). Enrichr: A comprehensive gene set enrichment analysis web server 2016 update. Nucleic Acids Research, 44(W1), W90–W97. 10.1093/nar/gkw377 27141961 PMC4987924

[acel13800-bib-0013] Lesnefsky, E. J. , Chen, Q. , & Hoppel, C. L. (2016). Mitochondrial metabolism in aging heart. Circulation Research, 118(10), 1593–1611. 10.1161/CIRCRESAHA.116.307505 27174952 PMC5009371

[acel13800-bib-0014] Lesnefsky, E. J. , Chen, Q. , Tandler, B. , & Hoppel, C. L. (2017). Mitochondrial dysfunction and myocardial ischemia‐reperfusion: Implications for novel therapies. Annual Review of Pharmacology and Toxicology, 57, 535–565. 10.1146/annurev-pharmtox-010715-103335 PMC11060135 27860548

[acel13800-bib-0015] Liu, L. X. , Rowe, G. C. , Yang, S. , Li, J. , Damilano, F. , Chan, M. C. , Lu, W. , Jang, C. , Wada, S. , Morley, M. , Hesse, M. , Fleischmann, B. K. , Rabinowitz, J. D. , Das, S. , Rosenzweig, A. , & Arany, Z. (2017). PDK4 inhibits cardiac pyruvate oxidation in late pregnancy. Circulation Research, 121(12), 1370–1378. 10.1161/CIRCRESAHA.117.311456 28928113 PMC5722682

[acel13800-bib-0016] Lopaschuk, G. D. , & Stanley, W. C. (1997). Glucose metabolism in the ischemic heart. Circulation, 95(2), 313–315. 10.1161/01.cir.95.2.313 9008441

[acel13800-bib-0017] Menendez‐Montes, I. , Abdisalaam, S. , Xiao, F. , Lam, N. T. , Mukherjee, S. , Szweda, L. I. , Asaithamby, A. , & Sadek, H. A. (2021). Mitochondrial fatty acid utilization increases chromatin oxidative stress in cardiomyocytes. Proceedings of the National Academy of Sciences of the United States of America, 118(34), e2101674118. 10.1073/pnas.2101674118 34417314 PMC8403954

[acel13800-bib-0018] Mohsin, A. A. , Chen, Q. , Quan, N. , Rousselle, T. , Maceyka, M. W. , Samidurai, A. , Thompson, J. , Hu, Y. , Li, J. , & Lesnefsky, E. J. (2019). Mitochondrial complex I inhibition by metformin limits reperfusion injury. The Journal of Pharmacology and Experimental Therapeutics, 369(2), 282–290. 10.1124/jpet.118.254300 30846619 PMC6474909

[acel13800-bib-0019] Pettersen, I. K. N. , Tusubira, D. , Ashrafi, H. , Dyrstad, S. E. , Hansen, L. , Liu, X. Z. , Nilsson, L. I. H. , Løvsletten, N. G. , Berge, K. , Wergedahl, H. , Bjørndal, B. , Fluge, Ø. , Bruland, O. , Rustan, A. C. , Halberg, N. , Røsland, G. V. , Berge, R. K. , & Tronstad, K. J. (2019). Upregulated PDK4 expression is a sensitive marker of increased fatty acid oxidation. Mitochondrion, 49, 97–110. 10.1016/j.mito.2019.07.009 31351920

[acel13800-bib-0020] Pilegaard, H. , & Neufer, P. D. (2004). Transcriptional regulation of pyruvate dehydrogenase kinase 4 in skeletal muscle during and after exercise. The Proceedings of the Nutrition Society, 63(2), 221–226. 10.1079/pns2004345 15294034

[acel13800-bib-0021] Ren, D. , Fedorova, J. , Davitt, K. , Van Le, T. , Griffin, J. H. , Liaw, P. C. , Esmon, C. T. , Rezaie, A. R. , & Li, J. (2022). Activated protein C strengthens cardiac tolerance to ischemic insults in aging. Circulation Research, 130(2), 252–272. 10.1161/CIRCRESAHA.121.319044 34930019 PMC8882057

[acel13800-bib-0022] RStudio Team . (2020). RStudio: Integrated development for R. RStudio, PBC. http://www.rstudio.com/

[acel13800-bib-0023] Satija, R. , Farrell, J. A. , Gennert, D. , Schier, A. F. , & Regev, A. (2015). Spatial reconstruction of single‐cell gene expression data. Nature Biotechnology, 33(5), 495–502. 10.1038/nbt.3192 PMC4430369 25867923

[acel13800-bib-0024] Shuai Zhang, M. W. H. , McMillan, R. P. , Cline, M. A. , & Gilbert, E. R. (2014). The pivotal role of pyruvate dehydrogenase kinases in metabolic flexibility. Nutrition and Metabolism, 11, 10. 10.1186/1743-7075-11-10 24520982 PMC3925357

[acel13800-bib-0025] Sithara, T. , & Drosatos, K. (2021). Metabolic complications in cardiac aging. Frontiers in Physiology, 12, 669497. 10.3389/fphys.2021.669497 33995129 PMC8116539

[acel13800-bib-0026] Tao, R. , Xiong, X. , Harris, R. A. , White, M. F. , & Dong, X. C. (2013). Genetic inactivation of pyruvate dehydrogenase kinases improves hepatic insulin resistance induced diabetes. PLoS One, 8(8), e71997. 10.1371/journal.pone.0071997 23940800 PMC3733847

[acel13800-bib-0027] Tim Stuart, A. B. , Hoffman, P. , Hafemeister, C. , Papalexi, E. , Mauck, W. M., III , Hao, Y. , Stoeckius, M. , Smibert, P. , & Satija, R. (2019). Comprehensive integration of single‐cell data. Cell, 177(7), 1888–1902. 10.1016/j.cell.2019.05.031 31178118 PMC6687398

[acel13800-bib-0028] Tran, D. H. , & Wang, Z. V. (2019). Glucose metabolism in cardiac hypertrophy and heart failure. Journal of the American Heart Association, 8(12), e012673. 10.1161/JAHA.119.012673 31185774 PMC6645632

[acel13800-bib-0029] Tsao, C. W. , Aday, A. W. , Almarzooq, Z. I. , Alonso, A. , Beaton, A. Z. , Bittencourt, M. S. , Boehme, A. K. , Buxton, A. E. , Carson, A. P. , Commodore‐Mensah, Y. , Elkind, M. S. V. , Evenson, K. R. , Eze‐Nliam, C. , Ferguson, J. F. , Generoso, G. , Ho, J. E. , Kalani, R. , Khan, S. S. , Kissela, B. M. , … Martin, S. S. (2022). Heart disease and stroke Statistics‐2022 update: A report from the American Heart Association. Circulation, 145(8), e153–e639. 10.1161/CIR.0000000000001052 35078371

[acel13800-bib-0030] Uhlén, M. , Fagerberg, L. , Hallström, B. M. , Lindskog, C. , Oksvold, P. , Mardinoglu, A. , Sivertsson, Å. , Kampf, C. , Sjöstedt, E. , Asplund, A. , Olsson, I. , Edlund, K. , Lundberg, E. , Navani, S. , Szigyarto, C. A. , Odeberg, J. , Djureinovic, D. , Takanen, J. O. , Hober, S. , … Pontén, F. (2015). Proteomics. Tissue‐based map of the human proteome. Science, 347(6220), 1260419. 10.1126/science.1260419 25613900

[acel13800-bib-0031] Wang, L. , Quan, N. , Sun, W. , Chen, X. , Cates, C. , Rousselle, T. , Zhou, X. , Zhao, X. , & Li, J. (2018). Cardiomyocyte‐specific deletion of Sirt1 gene sensitizes myocardium to ischaemia and reperfusion injury. Cardiovascular Research, 114(6), 805–821. 10.1093/cvr/cvy033 29409011 PMC5909650

[acel13800-bib-0032] Wei, X. , Shi, X. , Kim, S. , Patrick, J. S. , Binkley, J. , Kong, M. , McClain, C. , & Zhang, X. (2014). Data dependent peak model based spectrum deconvolution for analysis of high resolution LC‐MS data. Analytical Chemistry, 86(4), 2156–2165. 10.1021/ac403803a 24533635 PMC3982975

[acel13800-bib-0033] Wei, X. , Shi, X. , Kim, S. , Zhang, L. , Patrick, J. S. , Binkley, J. , McClain, C. , & Zhang, X. (2012). Data preprocessing method for liquid chromatography‐mass spectrometry based metabolomics. Analytical Chemistry, 84(18), 7963–7971. 10.1021/ac3016856 22931487 PMC3471364

[acel13800-bib-0034] Wei, X. , Sun, W. , Shi, X. , Koo, I. , Wang, B. , Zhang, J. , & Zhang, X. (2011). MetSign: A computational platform for high‐resolution mass spectrometry‐based metabolomics. Analytical Chemistry, 83(20), 7668–7675. 10.1021/ac2017025 21932828 PMC3196362

[acel13800-bib-0035] Wickham, H. (2016). *ggplot2*: *Elegant graphics for data analysis*. In *Use R!*, (pp. 1 online resource; XVI, 260 pages 232 illustrations, 140 illustrations in color). 10.1007/978-3-319-24277-4

[acel13800-bib-0036] Wu, Q. Q. , Xiao, Y. , Yuan, Y. , Ma, Z. G. , Liao, H. H. , Liu, C. , Zhu, J. X. , Yang, Z. , Deng, W. , & Tang, Q. Z. (2017). Mechanisms contributing to cardiac remodelling. Clinical Science (London, England), 131(18), 2319–2345. 10.1042/CS20171167 28842527

[acel13800-bib-0037] Xu, Z. , Alloush, J. , Beck, E. , & Weisleder, N. (2014). A murine model of myocardial ischemia‐reperfusion injury through ligation of the left anterior descending artery. Journal of Visualized Experiments, 86, 51329. 10.3791/51329 PMC4080806 24747599

[acel13800-bib-0038] Yuhan Hao, S. H. , Andersen‐Nissen, E. , Mauck, W. M., III , Zheng, S. , Butler, A. , Lee, M. J. , Wilk, A. J. , Darby, C. , Zager, M. , Hoffman, P. , Stoeckius, M. , Papalexi, E. , Mimitou, E. P. , Jain, J. , Srivastava, A. , Stuart, T. , Fleming, L. M. , Yeung, B. , Rogers, A. J. , … Satija, R. (2021). Integrated analysis of multimodal single‐cell data. Cell, 184(13), 3573–3587. 10.1016/j.cell.2021.04.048 34062119 PMC8238499

[acel13800-bib-0039] Zhang, H. , Yan, M. , Liu, T. , Wei, P. , Chai, N. , Li, L. , Wang, J. , Yu, X. , Lin, Y. , Qiu, B. , & Zhao, Y. (2022). Dynamic mitochondrial proteome under polyamines treatment in cardiac aging. Frontiers in Cell and Development Biology, 10, 840389. 10.3389/fcell.2022.840389 PMC8965055 35372351

[acel13800-bib-0040] Zhang, J. , He, Z. , Fedorova, J. , Logan, C. , Bates, L. , Davitt, K. , le, V. , Murphy, J. , Li, M. , Wang, M. , Lakatta, E. G. , Ren, D. , & Li, J. (2021). Alterations in mitochondrial dynamics with age‐related Sirtuin1/Sirtuin3 deficiency impair cardiomyocyte contractility. Aging Cell, 20(7), e13419. 10.1111/acel.13419 34216536 PMC8282250

[acel13800-bib-0041] Zhao, G. , Jeoung, N. H. , Burgess, S. C. , Rosaaen‐Stowe, K. A. , Inagaki, T. , Latif, S. , Shelton, J. M. , McAnally, J. , Bassel‐Duby, R. , Harris, R. A. , Richardson, J. A. , & Kliewer, S. A. (2008). Overexpression of pyruvate dehydrogenase kinase 4 in heart perturbs metabolism and exacerbates calcineurin‐induced cardiomyopathy. American Journal of Physiology. Heart and Circulatory Physiology, 294(2), H936–H943. 10.1152/ajpheart.00870.2007 18083902

